# The Use of Selected Machine Learning Methods in Dairy Cattle Farming: A Review

**DOI:** 10.3390/ani15142033

**Published:** 2025-07-10

**Authors:** Wilhelm Grzesiak, Daniel Zaborski, Marcin Pluciński, Magdalena Jędrzejczak-Silicka, Renata Pilarczyk, Piotr Sablik

**Affiliations:** 1 Laboratory of Biostatistics, Bioinformatics and Animal Research, West Pomeranian University of Technology, 71-270 Szczecin, Poland; 2 Faculty of Computer Science and Information Technology, West Pomeranian University of Technology, 71-210 Szczecin, Poland

**Keywords:** machine learning, data mining, artificial intelligence, dairy cattle, farming, performance indicators, classification, regression, supervised learning, unsupervised learning

## Abstract

The aim of this review was to present selected machine learning (ML) algorithms used in dairy cattle farming in recent years (2020–2024). A description of ML methods (linear and logistic regression, classification and regression trees, chi-squared automatic interaction detection, random forest, AdaBoost, support vector machines, k-nearest neighbors, naive Bayes classifier, multivariate adaptive regression splines, artificial neural networks, including deep neural networks and convolutional neural networks, as well as Gaussian mixture models and cluster analysis), with some examples of their application in various aspects of dairy cattle breeding and husbandry, is provided. In addition, the stages of model construction and implementation, as well as the performance indicators for regression and classification models, are described. Finally, time trends in the popularity of ML methods in dairy cattle farming are briefly discussed.

## Simple Summary

The current trend in animal husbandry, including cattle farming, is toward increasing stocking density and automating individual activities in animal care. Various electro-optical, acoustic, mechanical, and biological sensors provide very large amounts of information that become difficult to process in digital form. Multidimensional and highly complex datasets are characterized by non-linearity and relationships between many variables, which makes them hard to analyze using classical statistical methods. In this case, the so-called machine learning (ML) approach can be helpful. Therefore, this review presents the application of selected ML algorithms in dairy cattle farming during recent years (2020–2024), explaining their concepts and giving examples of their use in various aspects of cattle breeding and husbandry. In addition, the review briefly discusses the stages of model construction and implementation, the calculation and interpretation of basic performance indicators for regression and classification models, and the current trends in the popularity of ML methods applied to dairy cattle rearing.

## 1. Introduction

The current trend in animal husbandry is toward increasing stocking density and automating individual activities in animal care [1]. Various electro-optical, acoustic, mechanical, or biological sensors provide very large amounts of information that become difficult to analyze in digital form. Multidimensional and highly complex datasets are characterized by non-linearity and relationships between many variables, which makes their analysis using classical statistical methods challenging. Hence, the increasing use of machine learning (ML) alternatives, which provide well-suited methodologies for extracting knowledge from such data [2,3].

**Figure 1:**
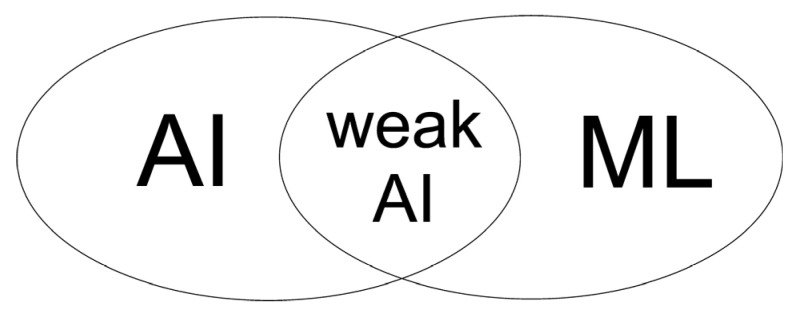
The relationship between AI and ML methods.

ML and artificial intelligence (AI) algorithms are two groups of methods, including techniques that can only be classified as belonging to one of them (see Figure 1). For example, AI encompasses various search and optimization techniques (such as genetic and evolutionary algorithms, particle swarm optimization, and others) or methods for uncertain information processing, such as fuzzy logic. Similarly, among ML algorithms, there are those that are not classified as AI, but rather as statistical methods (see the later part of this section).

On the other hand, many ML algorithms are classified as AI methods. However, they mostly belong to the field of the so-called “weak” AI, which deals with specific problems with well-defined goals and criteria for their achievement. This is the so-called computational intelligence that imitates living systems in order to solve problems and achieve predetermined goals [4]. We can also distinguish the so-called “strong” AI, which assumes the possibility of building a thinking system at the level of human intelligence or even surpassing it [5]. Most ML algorithms constitute a specific method within AI, allowing systems to learn from data and analyze them, thus automatically creating different analytical models. Due to their learning flexibility, they become the optimal selection for changeable data, requests, and tasks, or in cases where coding a solution is very difficult or impossible. Models developed by ML methods can adapt to variable conditions and improve their own performance over time [6].

Two main types of ML can be distinguished: supervised learning, in which the model learns from a set of data where each example corresponds to a known outcome, and unsupervised learning, in which the model learns without knowing the final outcome and tries to discover relationships in the data. This learning mode is particularly useful in data exploration for understanding their structure [7,8].

A model is developed (trained) on the training set, and some part of the data can be used for its testing. The created and trained model is verified on test data to determine its quality. The dataset used in ML includes conditional attributes and their corresponding outcomes or labels, i.e., decision attributes. Conditional attributes can be directly available, but it is often necessary to extract them from the input data using a variety of feature extraction methods [9]. Although the implementation of ML models is straightforward, some challenges exist in selecting appropriate algorithms, tuning parameters, and extracting features for better prediction accuracy [10].

Modern systems learn from vast amounts of diverse data. Due to iterations, continuous improvement in the accuracy and reliability of calculations is possible. Various ML algorithms are used for diagnosis, prediction, pattern recognition, rule extraction, classification, identification, and anomaly detection [11]. ML methods can be generally divided into supervised and unsupervised methods (both are described in this review). Supervised learning includes linear, polynomial and logistic regression, decision trees, artificial neural networks, k-nearest neighbors, naive Bayes classifier, random forest, support vector machines, and others. Unsupervised learning involves cluster analysis (k-means, hierarchical clustering) and neural networks [8,12].

ML is used in several overlapping areas of dairy cattle farming. In the field of health and welfare, by analyzing data from sensors attached to animals (collars, ear tags, pedometers), it becomes possible to detect early signs of diseases such as ketosis, mastitis, or lameness (e.g., by identifying movement patterns) [13]. Analysis of historical and current data allows for disease risk determination and improved prophylaxis, which consequently reduces the amount of antibiotics used and affects overall herd health [14,15]. Monitoring cows’ facial expressions can assist in detecting organismal malfunction [16].

In the area of productivity, various ML models are utilized to analyze genetic, nutritional, and environmental data, predict milk production for individual cows, and support a more precise selection of animals for further breeding. They also monitor milk parameters (fat, protein and lactose content, somatic cell count), enabling the detection of anomalies and indicating diseases or nutritional errors, which can be quickly corrected [17,18]. In the field of nutrition, feed composition, feed intake, and health parameters can be analyzed with the help of various models, tailoring diets to the individual needs of cows. Feeding efficiency can also be predicted, resulting in reduced costs and losses [19]. In the area of reproduction, the analysis of the physical activity and behavioral patterns of cows supports more precise ovulation detection, leading to a higher rate of successful inseminations [20,21,22].

In the domain of strictly genetic analyses like genomics, ML models are trained on large datasets of genotypes and phenotypes mainly to predict breeding values for specific traits, such as immunity, longevity, or productive life span, which ensures a more precise animal selection based on the highest genetic value and positively influences breeding decisions [23]. Many ML models have been successfully implemented to predict genomic breeding values in various animal species, including dairy cattle [24]. Trait selection techniques are crucial in genomic prediction, since they identify the most informative genetic markers, mainly single-nucleotide polymorphisms. However, it should be emphasized that ML models tend to overfit, and the selection of their optimal hyperparameters can be difficult in practice. Moreover, training datasets should be very large, especially for deep learning algorithms, and model interpretation can be problematic in genomic studies [25]. In developing countries, due to the lack of reliable phenotyping procedures and the recording of pedigree data, the development of genomic technologies can solve the problems of pedigree errors by replacing genomic relationship matrices with pedigree matrices [26]. The use of ML in genomic analyses is extensively presented in [27].

The application of ML models improves the management of the entire farm. By analyzing milk production, prices, and costs, it is possible to optimize milking and feeding schedules, implement the best economic strategies, and streamline other farm management processes [28].

Perhaps some readers are wondering about the difference between ML and statistical models. Why are we discussing linear or logistic regression as ML models, even though they have, after all, been typical statistical methods for a long time? It should be noted that the main difference between ML and statistical models is the idea of their application. In general, ML models are developed to obtain the most accurate predictions, while strictly statistical models are constructed to infer relationships and differences between variables [4]. Theoretically or technically, this statement is correct, but not completely precise, since we are always dealing with statistics and statistical models. A statistical model is a model for the data, developed to infer something about the relationships within the data or to create another new model capable of predicting future values (often the two go hand in hand). So, there are statistical models that can make predictions with varying degrees of accuracy, and there are ML models that in turn provide varying degrees of interpretation of the data (from highly interpretable regression models to “black-box” neural networks), but generally they sacrifice interpretability for predictive power.

ML is based on statistics, but also on many other fields (such as mathematics and computer science). Some differences between statistics and ML also lie in the fact that the former is based on probability spaces, and the latter is built on statistical theory, which is based on the axiomatic notion of probability spaces [29]. For example, we can train a linear regression model and obtain the same result as a linear (statistical) regression model aimed at minimizing the squared error between the data points. In one case, we perform “training”, i.e., we build a model using a subset of our data. We do not know how well the model will perform until we test it on the data that were not used during the training process (the test set). The aim of ML in this case is to obtain the best performance on the test set. In contrast, for a linear regression statistical model, we find a model that minimizes the mean squared error on all data, assuming they are linear. No training or testing is necessary in this case.

In many applications, the purpose of model building is to characterize the relationship between the data and the output (dependent) variable, and not to predict future data. This procedure is called statistical inference, as opposed to prediction [30]. But the model can also be used for prediction (which may be another purpose of its construction). However, the way the model is evaluated does not involve a set of tests, but just assessing the significance and robustness of its parameters. ML is about developing models that make repeatable predictions. Usually, their interpretability is not important, whereas statistical models are more about identifying relationships between variables, determining the significance of those relationships, and (additionally) making predictions. Proving a (statistically significant) relationship between variables requires the use of a statistical model, and not an ML model. Predictions can be important, but the lack of interpretability provided by most ML algorithms makes it difficult to prove relationships within the data [31].

Evaluation of an ML model involves a test set to verify (validate) its accuracy. In contrast, the analysis of the statistical model parameters using confidence intervals, significance levels, and other tests allows for assessing the validity of the model. Since these methods produce the same results, it is easy to understand why they can be mistakenly assumed to be the same.

In traditional statistical methods, the concept of training and testing is non-existent, but the model quality is evaluated with appropriate indicators. This procedure is different from that used in ML, but both methods are able to give statistically sound results. The traditional statistical approach provides an optimal solution, since this solution has a closed form. In ML, several different models are investigated to converge on the final hypothesis, which happened to be consistent with the results of the regression algorithm. If a different loss function was used, the results would not converge. This creates a certain discomfort of nontransparency and black-box confinement in the sense that the ever-increasing complexity of ML algorithms effectively prevents users from understanding the way they work [32].

## 2. Characteristics of Selected ML Models

Below, we present selected ML models with their brief characteristics.

### 2.1. Linear Regression (LR)

LR is an ML technique for evaluating the effect of a number of different features (called independent variables, explanatory variables, or predictors) on some feature of particular interest (called the dependent, explained, or response variable), appearing in continuous form [12,33].

The general linear regression formula can be written in matrix form [34]:

(1) y=Xβ+ε,

where ***y*** is the vector of the dependent variable, ***X*** is the matrix of independent variables, ***ε*** is the error vector (for independent variables with normal distribution *E*(***ε***) = **0** and covariance matrix *Var*(***ε***) = *σ^2^* ***I***), and ***β*** is the vector of regression coefficients.

When using this technique, it is important to remember that the independent variables should have additive effects on the values of the dependent variables, and the residuals of the model (errors) should be characterized by homogeneous variance (homoscedasticity), normal distribution, and lack of autocorrelation, which is not always satisfied in practice [33,35,36,37]. The regression model is able to use large amounts of data and make quick predictions; however, it is prone to over-fitting and consequently poor generalization [38,39].

There are several types of regression algorithms such as linear regression, which assumes a linear relationship between the input features and the predicted value, and nonlinear regression, which allows the representation of more complex relationships between inputs and outputs [40]. Polynomial regression is a special case of regression that makes it possible to predict values of numerical variables based on other variables, while taking into account the non-linear relationships between them [41]. In this regression method, it is assumed that the relationships between variables can be represented by polynomials of various degrees (above unity). The analyzed data must be transformed into a format appropriate for the ML algorithm, and the optimal degree of polynomial must be selected [42]. The model is developed on the basis of the training data, which contains information about the explanatory variables and the corresponding values of the target variables. Optimal regression coefficients are selected using various optimization algorithms, e.g., the least squares method or gradient algorithms [43]. The model is evaluated on test data, so that its generalizability can be verified. It should be emphasized that a too high polynomial degree can result in over-fitting, leading to inaccurate model predictions [44].

Linear regression is an excellent tool for analyzing associations between variables, especially when the relationship between the independent and dependent variables is linear; however, it is not recommended for most practical applications since it oversimplifies real-world problems by assuming a linear relationship between variables [45]. An example of the use of linear regression in dairy cattle farming is the study on the relationship between protein content and milk production, in which the former was predicted based on total milk yield and the best-fitting model for this purpose was selected from among different types of regression models, such as power, quadratic, or cubic [40]. On the other hand, Korean researchers applied a regression model to study the effect of heat stress on milk traits using comprehensive data analysis (including dairy production and climatic factors). A segmented regression model was developed to estimate the effect of temperature and humidity index on milk traits and determine the optimal breakpoint value. It was observed that the milk production parameters decreased dramatically after a certain breakpoint was achieved, while the urea level and somatic cell count increased [46]. Finally, Nehara et al. [47] used multiple regression and artificial neural networks to predict the 305-day milk yield in the first lactation.

### 2.2. Logistic Regression (LogR)

In LogR, the explained variable must be dichotomous and follow a Bernoulli distribution [48,49]. It is assumed that the measurement errors are non-existent, which means that outliers strongly affect the final result. In addition, it is advisable to eliminate correlated features to avoid overfitting [33]. The LogR model takes real inputs and predicts the probability of class membership (to one of the two classes: 0 or 1). It calculates the probability of a variable *Y* taking a distinguished value (e.g., 1), conditional on the specific values of the explanatory variables (*x*_1_, *x*_2_, …, *x_k_*) [50,51]:

(2) $$P(Y=1|x_{1},\;x_{2},\ldots ,\;x_{k})=\frac{e^{z}}{1+e^{z}},$$

where *e* is the base of the natural logarithm, and *Z* is the multiple regression equation:

(3) $$Z=\beta_{0}+\sum_{i=1}^{k}\beta_{i}X_{i},$$

where *x*_1_, *x*_2_,…, *x_k_* are the independent variables and *β*_0_, *β*_1_,…, *β_k_* are the regression parameters.

A type of LogR is multinomial logistic regression, used when the dependent variable takes on more than two values. It is also known as softmax regression, multinomial logit, multiclass LogR, maximum entropy classifier (MaxEnt), or conditional maximum entropy model. The softmax function converts raw outputs (logits) into probabilities [52]. The types of LogR also include ordinal logistic regression (for multiple ordered classes, such as ranks or categories), alternating LogR for repeated measures, and others [53].

An example of the application of LogR in dairy cattle farming can be found in [54], in which difficult calvings were predicted using LogR, naive Bayes classifier, random forest, and decision trees. In addition, a sampling method was applied due to unbalanced data, which improved predictive performance (the F-measure for LogR was 0.426 on a balanced dataset). On the other hand, Zhou et al. [55] proposed an LogR model to predict metritis, mastitis, lameness, and digestive disorders in cows on the basis of their physical activity and rumination time combined with milk yield. It was concluded that milk production, physical activity, and rumination time could be used to identify these disorders early and automatically.

### 2.3. Multivariate Adaptive Regression Splines (MARS)

The MARS algorithm is useful in regression problems [56]. In this method, after taking into account the influence of individual explanatory variables, all observations of a given explanatory variable are analyzed, and the predictor variable space is divided into intervals in which the effect of this variable on the dependent variable differs [57]. The explanatory variable is included in the model (based on the so-called basis function) with different weights and signs, depending on whether its value is below or above a certain threshold [50,58]. In addition, this method allows for interactions between explanatory variables, which results in a better fit to the given set of factors affecting the phenomenon under study [50]. In general, the form of the MARS function is obtained by summing the *M* basis functions and products of these functions with appropriate weights [50,58,59,60,61]:

(4) $$f\left(x\right)=\alpha_{0}+\sum_{m=1}^{M}h_{m}(x),$$

where *h_m_*(***x***) is the tensor product of the splines (i.e., a basis function).

In order to obtain a good fit of the model to the empirical data, it is necessary to select a certain number of appropriate basis functions and determine the optimal number and position of the so-called knots. In subsequent iterations of the MARS algorithm, these procedures are carried out automatically [62]. In the initial stage of model construction, the maximum number of basis functions is included. These functions are subsequently eliminated from the model by an appropriate procedure, so that the quality of the model fit [measured by the generalized cross validation (*GCV*) error] is not too low [50,57,58,59]:

(5) $$GCV=\frac{\sum_{i=1}^{N}{\left(y_{i}-f(x_{i})\right)}^{2}}{{\left(1-\frac{M+d\cdot (M-1)/2}{N}\right)}^{2}},$$

where *N* is the number of cases in the dataset, *M* is the number of independent basis functions, *d* is the penalty coefficient for adding another basis function to the model, *f*(*x_i_*) is the MARS prediction, *y_i_* is the real value, and (*M* − 1)/2 represents the number of knots.

**Figure 2:**
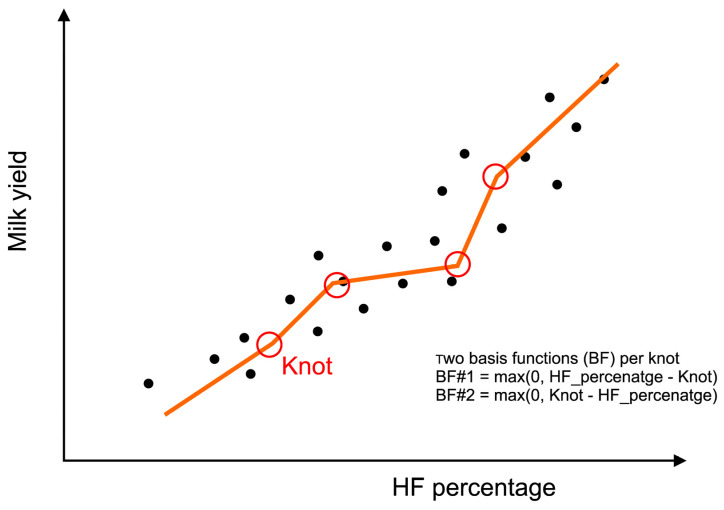
An example of the MARS model for milk yield prediction.

MARS, whose main idea is depicted in Figure 2, is a non-parametric procedure that makes no assumptions about the type of relationship between the dependent and independent variables [57]. It is particularly useful for higher-dimensional feature spaces (with more than two input variables) and very complex non-monotonic relationships, which are difficult to model by parametric methods [63]. An example of the application of this model in dairy cattle farming can be found in [64], in which support vector machines, elastic net regression, partial least squares regression, random forest, and MARS were used to predict lameness in cows. The differences in urinary metabolomics profiles at calving (transition period) and the time of lameness detection were evaluated to determine their usefulness in lameness prediction at an early stage (before and after gait changes). The final model accuracy was 82% (about 81% for MARS). In another study [65], different ML models were developed to predict subclinical mastitis in dairy cows based on potential predictors such as lactation number, days in milk, chromatic parameters (L, a, b, H, C), milk fat, protein and lactose content, milk freezing point and density, solids-not-fat, somatic cell count, pH, and electrical conductivity. Of the models used [classification and regression trees (CART), chi-square automatic interaction detection (CHAID), exhaustive CHAID, quick unbiased efficient statistical trees, and MARS], CART and MARS yielded the best results in correctly distinguishing between healthy and diseased cows.

### 2.4. Naive Bayes Classifier (NBC)

NBC is a method based on Bayes probability [66,67], which describes the probability of an event occurring based on prior knowledge of conditions that may be associated with the event [68]. NBC is a simple approach compared to most other ML methods, since it “naively” assumes complete independence between input variables [69,70].

Studies have shown that violating the independence assumption does not necessarily result in poor model performance [71,72]. The NBC achieves reasonable classification accuracy in practice despite its simplicity and is considered one of the most efficient ML algorithms in terms of computational speed and resource utilization. This makes the NBC suitable for large datasets, especially in practical applications [73,74,75].

The task of the NBC is to assign a new case to one of the decision classes, whose set must be finite and defined a priori. Each training case is described by a set of conditional attributes and one decision attribute [76]. If the distribution of the independent variables for each class is known, the probability of an observation [for which the independent variables take certain values (*x*_1_, *x*_2_, …, *x*_n_)] belonging to a particular class (*A*) is proportional to the value of the density function for that class with the values of the predictors (*x*_1_, *x*_2_, …, *x*_n_) multiplied by the a priori probability of class membership [77]. The a priori probability of class membership is usually assumed to be equal to the proportion of this class in the sample. An object (a case) is assigned to the class by simply selecting the one with the highest probability estimated from the above formula [68]. NBC adopts some simplifications (hence the name “naïve”): the predictors are not related to each other within the class (they are independent), which usually does not hold in practice, but allows the density function to be presented as the product of one-dimensional density functions for individual conditional attributes:

(6) $$f^{A}\left(x_{1},\;x_{2},\ldots ,x_{n}\right)=\prod f^{A}(x_{i}).$$

The type of distribution (usually normal) is assumed in advance, and only its parameters (mean and standard deviation) are estimated for each class [78].

The assumption of such “naivety” has an important mathematical value, as it allows the probability of the product of events to be replaced by the product of probabilities [68]. This has significant computational consequences, which make the implementation of NBC possible and enable the analysis of a large number (hundreds or even thousands) of variables. The NBC does not suffer from the so-called curse of dimensionality (when the correct classification of objects from the full dataset is almost impossible). As the number of input variables increases, computational and memory complexity scales linearly, not exponentially [79].

### 2.5. Support Vector Machine (SVM)

Between 1995 and 1998, Vladimir Vapnik [80,81] developed the concept of an SVM for classification problems. These classifiers, due to their greater generalizability, produce better results than, e.g., neural networks, and are less prone to overfitting [82]. SVMs belong to the group of methods that learn from training data. Unlike other solutions, the SVM treats them selectively, focusing on the most relevant ones, i.e., located around the class boundary [83]. The input is a set of pairs (***x***, *y*), with ***x*** being the attribute vector and *y* being the class label. SVM training involves identifying the position of a hyperplane separating the classes with the largest possible margin (distance) between them [48,84]. The plane is determined using the data lying on the class boundaries (the so-called support vectors). In the case of linear classification, the algorithm fits a linear function, and the points closest to the plane are called vectors. The fewer the number of support vectors, the greater the model’s ability to generalize.

After the training process is completed, the determined support vectors allow for classifying the data into their corresponding classes [85]. SVMs are most often used for binary classification, but multi-class implementations also exist. The disadvantage of SVMs is a decrease in performance with large and noisy datasets, which results in increased learning time. An advantage is their ability to deal with partially structured data and the possibility of their application in multiple classification tasks using complex (polynomial, radial, hyperbolic tangent) functions [83].

### 2.6. Decision Trees

Decision trees are an important tool in the field of AI and ML, and they are applied to classification and regression problems. They consist of a set of decision nodes connected by branches extending downward from the root node to the terminating leaf nodes [86]. There are several types of trees, a brief description of which is presented below.

Classification and regression trees (CARTs) are decision tree models in the form of tree-structured graphs that represent all possible decisions and their corresponding outcomes [48]. The tree is formed by recursively dividing a set of observations into *n* disjoint subsets. The idea is to obtain maximally homogeneous subsets in terms of the observed variable [87]. Starting from the root node, observations are partitioned into two disjoint subsets [35,88]. The divided set is the parent node, and the resulting subsets are the child nodes. The child node is subsequently split into smaller subsets until further division is impossible. A node without outgoing edges is called a leaf node and indicates the size of the tree [58]. The depth of the tree is the number of edges between the root and the most distant leaf [48]. The CART splitting rules include the Gini index and entropy measures [89]. The CART algorithm is suitable for the combinations of continuous and nominal variables, complex-structure data sets, outliers and missing data. It uses the same variables in different parts of the tree [68,90]. To prevent excessive tree growth, pruning is applied, which simplifies the tree structure and increases its generalizability [35,58].

Chi-squared automatic interaction detection (CHAID) is a type of decision tree technique that classifies a population into subgroups, so that the variation of a dependent variable is minimized within the groups and maximized among them [35,87]. This algorithm is a multivariate analysis technique that identifies the size and rank of statistically significant differences. In CHAID analysis, a chi-square test is used to determine the next best split at each step for nominal dependent variables, the likelihood-ratio test is performed for ordinal response variables, and the F-test is applied for continuous dependent variables [91]. The difference significance is evaluated with the *p*-value obtained from the test and compared to a predetermined significance level α [35,58]. The predictor with the smallest *p*-value is subsequently selected, producing the most significant split. This procedure is repeated until a subset (or subgroup) can no longer be divided due to the small sample size [92].

Two important strategies for increasing the accuracy of predictive models include boosting and bagging (which themselves are not ML models in the strict sense). They are based on merging a number of simple and less accurate models (with lower predictive performance) into one comprehensive and more accurate model. Each simple model is trained to correct the errors of the previous one, gradually increasing the overall performance of the whole ensemble. Boosting is most often applied to decision trees, which may not have high predictive power individually, but their combined accuracy improves significantly by merging many of them [93]. The most popular boosting algorithm is Adaboost (i.e., adaptive boosting), whose training begins by taking *M*-labeled training cases *S* = [(*X*_1_, *y*_1_), …, (*X_M_*, *y_M_*)], where *x_i_* belongs to some space *X*, being represented by a vector of input values, and *y_i_* is the labeled output associated with *x_i_*. The boosting algorithm is repeated in a series of rounds *t* = 1, …, *T*, during which increasingly higher weights are assigned to those cases that previously produced errors.

The weights for misclassified observations are determined according to the following formula:

(7) $$w_{i}\left(2\right)=w_{i}\left(1\right)\frac{1-b(1)}{b(1)}.$$

The weights for correctly classified cases do not change [i.e., *w*(2) = *w*(1)], and *b*(1) denotes the fraction of misclassifications for the first model. Subsequently, the model is fitted to the data with adjusted weights *w_i_*(2). The component models are combined by calculating their weighted sum:

(8) $$y_{i}=sign\left[\sum_{j}M_{j}\left(x_{i}\right)log\frac{1-b(j)}{b(j)}\right],$$

where *M_j_*(*x_i_*) denotes successive simple classification models obtained for the weights determined, as described above. In the case of binary classification, the functions *M_j_*(*x_i_*) take the values 1 and −1. When the error on the test set stops decreasing, no more component models are added. The final result of the boosting algorithm is a combination of all weak models with a weight determined according to their importance. The more accurate the model, the higher the weight. The resulting combination is a kind of “majority vote,” and the ultimate model is based on the weighted votes of the weak models [94].

Boosting is effective in reducing both random variation (variance) and systematic errors in predictions. It also has the unique feature of focusing on more difficult examples, based on the performance of poorer models. As a result, boosting algorithms perform better than other methods, such as bagging, being less sensitive to changes in the training data at the same time [94]. In addition to Adaboost, the gradient boosting algorithm (such as XGBoost), which builds models iteratively and minimizes errors during model development, is also used. Bagging (bootstrap aggregating), on the other hand, trains multiple models simultaneously (using random training subsets) and averages their predictions. Training models in parallel on different data samples increases their diversity, contributes to reduced variance, and avoids over-fitting. Such ensemble models are also less sensitive to outliers, as random sampling reduces their impact on model performance [95].

**Figure 3:**
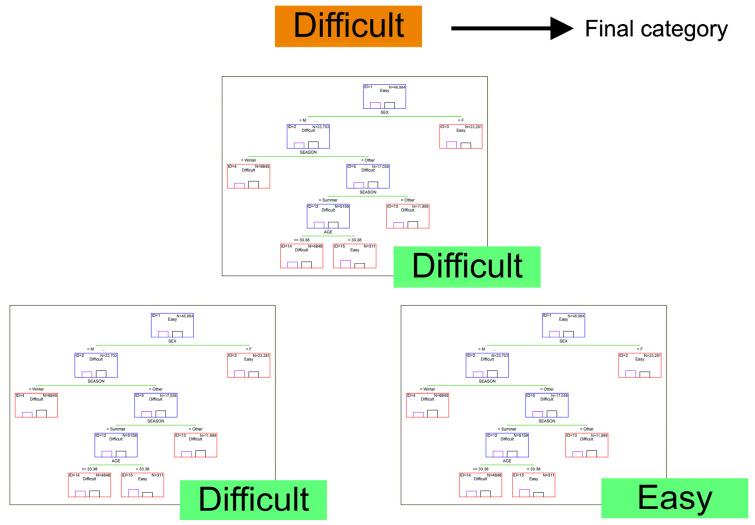
An example of dystocia prediction using RF.

Random forest (RF) is a type of bagging in which samples are taken randomly from the training set. However, unlike bagging, where a full set of features is provided to each tree, their random subsets are used to train individual trees [96]. Due to the random selection of features, the trees are more independent of each other compared to regular bagging, which often results in their higher predictive performance (associated with a better variance-bias tradeoff) and faster model development, since each tree only learns from a subset of the features [97]. A RF can be viewed as a collection of multiple decision trees with random sampling, aiming to eliminate the drawbacks of the basic decision tree algorithm. RF can reduce the instability of single decision trees and their tendency to overfit the training data by averaging the predictions obtained from many such trees [95]. It was successfully applied to estrus detection in Holstein × Gyr heifers, with sensitivity ranging between 73.3% and 99.4% [98]. The main idea behind RF is shown in Figure 3.

**Figure 4:**
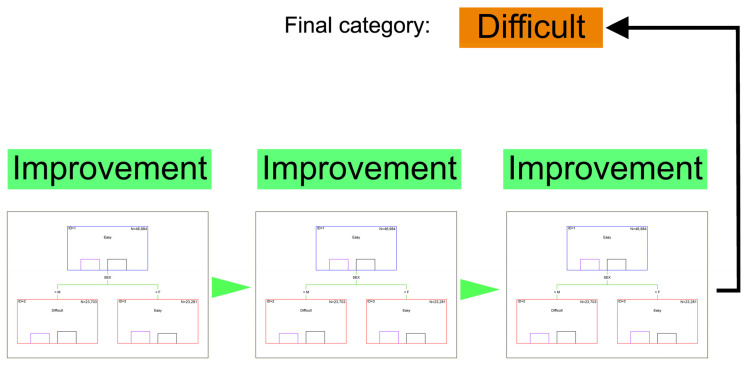
An example of dystocia prediction using BT.

Boosting creates an ensemble model by sequentially combining several weak decision trees into one strong classifier. Boosted trees (BTs) have begun to be applied relatively recently in prediction tasks as one of the most effective data mining methods. The use of additive weighted expansion of very simple trees can provide an almost perfect match between predicted and observed values, even if the modeled relationship between the predictors and the predicted variable is very complex [97,99,100]. BTs often outperform other classification models, such as neural networks, being particularly useful for detecting anomalies in supervised learning tasks on highly imbalanced datasets (e.g., low incidence of dystocia in cows) [101,102]. Both RF and BT are based on multiple decision trees used in ensemble modeling, which involves a larger number of simpler models applied simultaneously to achieve better prediction performance than with a single model [103,104,105]. This idea (boosting in particular) is considered one of the most useful and important statistical research results of the last two decades [106]. The main concept of BT is shown in Figure 4.

### 2.7. Artificial Neural Network (ANN)

**Figure 5:**
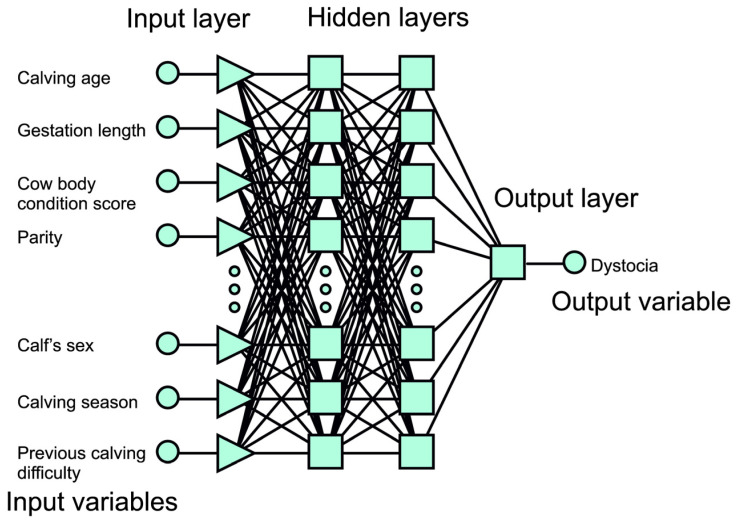
An example MLP for dystocia prediction.

ANNs are computational systems consisting of many individual processing units called artificial neurons, which function similarly to biological cells in the human brain [58,88]. Such networks are built from the layers of neurons. The input layer inputs data, which, once processed, are presented to subsequent layers. The use of appropriate training algorithms allows the network to recognize hidden patterns and correlations within the raw data, to group and classify them, and to learn and improve itself when new data are available [50]. The very popular multilayer perceptron (MLP) is a multilayer feedforward network in which all layers are fully connected [35,107]. Each neuron of a layer is connected to the neurons of an adjacent layer, and information flows in one direction. In other words, there are no intra-layer or supra-layer connections. MLP have been found to be efficient and simple to train; however, they are prone to over-fitting [108]. Even a reduction in the number of neurons in successive layers does not necessarily decrease the large amount of computing power required to train the network. Recently, so-called deep neural networks (DNNs) have emerged as models capable of recognizing complex patterns in raw data [109]. An example structure of MLP is shown in Figure 5.

**Figure 6:**
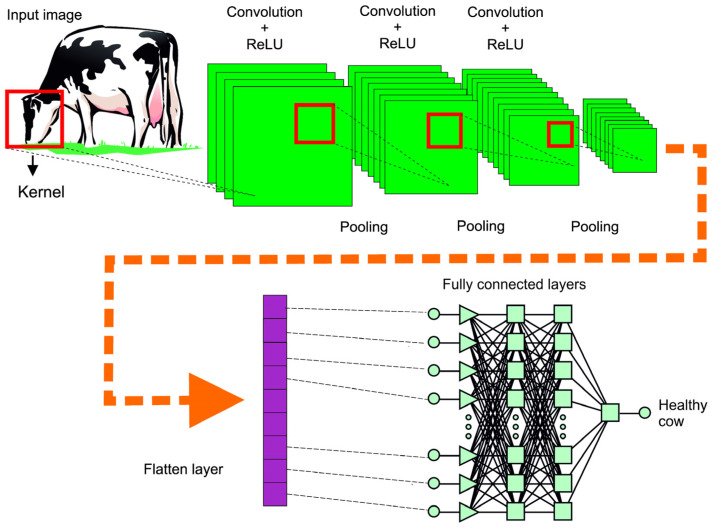
An example DNN for health status prediction (cow image by Dominique Benoist from Pixabay).

The structure of the DNN also consists of layers of connected neurons, but their number is higher than in the MLP-type networks. Each neuron is connected to those in adjacent layers by weights that reflect the strength and direction of the (excitatory or inhibitory) connection [108]. DNN models are characterized by their depth, size, and width. The number of layers contained in a DNN, excluding the input layer, is called its depth. The total number of neurons in the model is known as its size. Finally, the width of the DNN is the layer that includes the largest number of neurons [27]. An example DNN for health status prediction is shown in Figure 6.

When training a DNN, a set of observations (cases) is fed to an input layer. These observations serve as the input and output of that layer. In the hidden layers, each neuron receives a weighted sum of the outputs of the neurons from a layer at a lower level of the hierarchy and passes it through an activation function to determine its output. The most commonly used activation functions in the hidden layers are ReLU (rectified linear unit), hyperbolic tangent, and sigmoid function [110]. In the output layer, the DNN is supposed to perform classification or regression based on the nature of the target variable. In classification tasks, the number of neurons in the output layer is equal to the number of classes. In addition, different activation functions can be utilized depending on the type of target variable. For example, softmax is applied to nominal variables, and the sigmoid function is utilized for binary classification [108,109]. In regression problems, linear activation functions are used, and the output layer represents the estimated values of the target variables. The most effective activation function for a continuous variable is ReLU [111], while the tanh activation function (typically used in hidden layers) introduces nonlinearity in the DNN model. Being centered around zero (unlike the sigmoid function), it allows the network to learn from both positive and negative weights.

Like other ML models, DNN training involves determining optimal weights that minimize the difference between the actual and estimated values of the target variable. The gradient descent method is used to minimize the loss function. Weights must be adjusted during the learning process. When the DNN is being trained for the first time, these parameters are initialized randomly. Once observations are fed into the model, the information is propagated forward through the network until a specific output value is predicted. The gradients of the loss function are subsequently calculated using a hyperparameter called the learning rate (*η*), which indicates the magnitude of gradient descent steps and updates the function parameters (weights and deviations) for the neurons in the hidden layers. Back propagation is a method for calculating gradients [35,58], whose concept is based on the fact that the contribution of each neuron to the loss function is proportional to the weight of its connection to the neurons of the next layer. Therefore, these contributions can be calculated starting from the output layer and back-propagated through the network using the weights and derivatives of the activation function [108,109,112].

Deep learning covers a wide range of architectures. The most popular are feedforward networks, also known as MLP, recurrent neural networks (RNNs), and convolutional neural networks (CNNs). CNNs are designed to work with data represented as multiple arrays. The input variable can be one-dimensional, two-dimensional (such as color images), or three-dimensional (in the case of videos or computed tomography images) [113]. The architecture of a CNN consists of convolutional and pooling layers, followed by fully connected neural networks [112]. When training a CNN, the first two types of layers (convolutional and pooling) perform feature extraction. A fully connected neural network is supposed to perform classification or regression tasks. In the convolutional layer, a mathematical operation generates one filtered version of the original input data matrices. This convolutional process is called a “kernel” or “filter.”

A nonlinear activation function, typically ReLU, is applied after each convolution to produce results organized as feature maps. The pooling operation follows the smoothing of the results; its role is to merge semantically similar features into one. In other words, pooling reduces the number of parameters and makes the network less computationally expensive. The output of a fully connected neural network is passed to another activation function to perform classification or regression tasks based on the nature of the output variable [114]. A CNN has been successfully applied to facial image recognition of dairy cows [115], behavioral analysis [116], image-based body condition score estimation [117], tuberculosis prediction from milk spectral data [118] and various classification tasks [112,113,114].

### 2.8. Cluster Analysis (CA)

CA (clustering) is a set of methods for identifying homogeneous subsets of objects (cases) from the population. The idea is to separate them into a certain predetermined (or not) number of groups (clusters) of “similar” objects (cases), which at the same time are not similar to the objects in other groups (clusters). The key concept in CA is similarity, which can be defined as a function that assigns a real number to a pair of objects. The most commonly used functions include Chebyshev, Euclidean, squared Euclidean, and city-block (Manhattan) distances. Clustering can be hard (each case is assigned to a single cluster) or fuzzy (individual cases can occur in more than one cluster). CA can be divided into hierarchical and *k*-means clustering methods. The former is based on an agglomerative procedure, which merges objects into increasingly larger clusters, and a divisive approach, which separates them into smaller clusters [119]. In agglomerative methods, the analysis starts with a certain number of possible subsets (*n*) of cases. At each step, two possible subsets are combined, which in turn reduces the number of successive subsets (*n* − 1 → *n* − 2) until only one set or group is formed. During this process, two grouped cases remain permanently merged [120]. The actual grouping of cases is based on similarity and distance measures. The optimal number of clusters is usually determined after the merging process is completed.

The *k*-means method involves moving objects (cases) from cluster to cluster until the variability within clusters (maximum similarity of observations) and between clusters (maximum difference between clusters) is optimized. However, the number of clusters must be arbitrarily determined in advance (as this is a non-hierarchical method). The initial cluster centers (centroids) are randomly selected (e.g., as *k* observations or the first *k* observations), and the distances (Euclidean, squared Euclidean, Chebyshev, or other) to the centroids are calculated. Subsequently, the objects are assigned to the clusters (by comparing the distances of the observation from all clusters and assigning them to the one whose center is the closest), and the new centroids are determined, e.g., based on the arithmetic averages of the points belonging to each cluster. The algorithm usually terminates in the absence of object transfers between clusters or after reaching the maximum number of iterations set at the beginning [121].

### 2.9. k-Nearest Neighbor (k-NN)

One of the most basic methods used for classification and regression is the k-NN algorithm. It belongs to the domain of supervised learning and is widely applied in pattern recognition, data mining, and anomaly detection.

The basic assumption of the nearest neighbor method is that similar observations (cases) are close to each other, and outliers are usually isolated and distant from the cluster of similar observations. The appropriate choice of parameter *k* depends on the type of data. On the one hand, the larger the *k* value, the less influence the noise present in the data has on the classification results. On the other hand, a high value of *k* makes the boundaries between classes less distinct [122].

### 2.10. Gaussian Mixture Model (GMM)

GMMs are probability density models used to analyze and cluster data. GMMs consist of a mixture of one or more multivariate normal distributions and represent the probability density distribution of a set of data points [123]. They constitute a generalization of the k-means algorithm (a hierarchical approach). The clustered data points are not labeled with the predicted values but are expressed as a Gaussian mixture, where each component represents a single variable. Each mixture is a probability density function that defines the probability of the data values under a particular distribution. The model assigns a probability to each cluster, i.e., the probability of a data point belonging to that cluster. GMMs can identify clusters in data that contain multiple overlapping distributions. They are flexible and have the ability to model complex data, but require large datasets to make accurate predictions, and the number of components included in a model can affect its accuracy and performance [124].

An example of the use of GMMs in dairy cattle farming is cow gait type recognition based on a GMM and a hidden Markov model. Sensor data were pre-processed (denoised and restored to real dynamic values) and clustered to serve as inputs for the hidden Markov model. The recognition of gait types (stationary, standing, and swing) was accomplished by decoding the observed data. The presented method may serve as the basis for lameness detection in dairy cows [125].

### 2.11. Quality Assessment of Models

During the model development stage, data are usually divided into three subsets: a training set (*n_L_*), a smaller validation set (*n_w_*), which is used to control the training process, and a test set (*n_T_*), which verifies the model’s predictive performance. Different types of approximations are used to determine the size of each set [126]. Ivachnienko and Jurackovskij [127] proposed the following division:

(9) $$n_{L}=n_{T}+\frac{n}{2},$$

where *n_L_* is the size of the training set, *n_T_* is the size of the test set, and *n* is the total sample size.

However, it is quite common to determine the size of these sets arbitrarily in the proportions of 70%, 15%, and 15% (or others such as 40%, 40%, and 20%, or 50%, 25%, and 25%) for the training, validation, and test set, respectively. In general, the training set should be reasonably large to ensure the representativeness of the phenomenon under study. Another method of model quality assessment is *k*-fold cross-validation, in which the entire dataset is randomly divided into *k* (e.g., 10) approximately equal subsets, of which *k*-1 are used to train a model, and one (the *k*th) serves as an independent test set. This procedure is repeated *k* times. As a result, each part (subset) of the original dataset is used as a test set exactly once, and each of the *k* iterations generates a separate predictive model (e.g., a single tree or ANN). In the final step, the prediction quality of the *k* models is averaged [128]. An example of the use of 10-fold cross-validation can be found in [129], in which new traits in dairy cattle were predicted using sensor data (dry matter and residual feed intake based on milk spectral analysis).

The developed models can be evaluated using the various criteria presented below.

### 2.12. Quality Measures for Regression Models

In order to assess the quality of regression models, the following indicators are mainly used: the Pearson correlation coefficient between the observed and predicted values (*r*), the ratio of the standard deviation of the error term to that of the dependent variable (*SD_ratio_*), and the error standard deviation (*SE*) [58]. The smaller the *SD_ratio_* value, the better the model quality. A very good model obtains values ranging from 0 to 0.1. *SD_ratio_* greater than unity indicates poor model quality [35,88]. In the case of Pearson’s correlation coefficient, the values range from 0 to 1. The higher the value, the better the model. The error standard deviation for a good model should be as small as possible. Other predictive performance measures include [35,58,130,131,132,133]:

1. Relative prediction error (*E*):
  (10) $$E=\frac{y_{i}-\hat{y}}{y_{i}},$$
2. Mean prediction error (*ME*):
  (11) $$ME=\frac{1}{n}\sum_{i=1}^{n}\left(y_{i}-\hat{y}\right),$$
3. Mean absolute prediction error (*MAE*):
  (12) $$MAE=\frac{1}{n}\sum_{i=1}^{n}\left|y_{i}-\hat{y}\right|,$$
4. Global relative approximation error (*RAE*):
  (13) $$RAE=\sqrt{\frac{\sum_{i=1}^{n}{\left(y_{i}-\hat{y}\right)}^{2}}{\sum_{i=1}^{n}{y_{i}}^{2}}},$$
5. Mean squared error (*MSE*), which is the mean square of the differences between the actual and predicted values:
  (14) $$MSE=\frac{1}{n}\sum_{i=1}^{n}{\left(y_{i}-\hat{y}\right)}^{2},$$
6. Root mean squared error (*RMSE*):

(15) $$RMSE=\sqrt{\frac{1}{n}\sum_{i=1}^{n}{\left(y_{i}-\hat{y}\right)}^{2}},$$

where *y_i_* is the real value, $\hat{y}$ is the value determined by the model, and *n* is the number of observations.

One can also use the coefficient of determination (*R*^2^) or the adjusted coefficient of determination ($R_{p}^{2}$) [87]. *R*^2^ determines the proportion of the variation in the dependent variable under study accounted for by the predictors included in the model. The higher the value, the better the model. *R*^2^ below 0.4 indicates poor model quality [134].

*R*^2^ shows the percentage by which the model predictions are closer to the observed values compared to the average predicted value. The closer to unity, the more the variance in the value of the predicted variable is explained by the variables included in the model [88]:

(16) $$R^{2}=\frac{\sum_{i=1}^{n}{\left(\hat{y}-\bar{y}\right)}^{2}}{\sum_{i=1}^{n}{\left(y_{i}-\bar{y}\right)}^{2}},$$

$R_{p}^{2}$, which can be used to compare several models (with different numbers of predictors), is estimated according to the following formula [134,135]:

(17) $$R_{p}^{2}=1-\frac{{MS}_{E}}{{MS}_{T}},$$

where *MS_E_* is the estimated variance of the model error and *MS_T_* is the estimated total variance.

### 2.13. Quality Measures for Classification Models

In classification issues, a classification (confusion) matrix is often presented for two predicted classes conventionally defined as a positive and negative class (i.e., binary classification), such as the occurrence or non-occurrence of a disease (Table 1). This matrix summarizes all cases (elements) correctly and incorrectly classified by a model [136].

In Table 1, true positive (*TP*) is the number of cases that actually belong to the positive class (e.g., sick cows, those belonging to one of the two breeds or having a characteristic trait, etc.) and were correctly classified into this class by the model; false negative (*FN*) is the number of cases belonging to the positive class, but incorrectly classified into the negative class by the model; true negative (*TN*) is the number of cases belonging to the negative class (e.g., healthy cows, those belonging to another breed or animals that do not have a characteristic trait) and correctly classified into this class by the model, and false positive (*FP*) is the number of cases belonging to the negative class, but incorrectly classified into the positive class by the model [74,137].

Such a matrix serves as the basis for determining various measures that describe the model classification ability [138]. Typically, researchers determine which individuals belong to the positive and negative classes based on their earlier premises. In the case of animal diseases, ill individuals are usually assigned to the positive class, and healthy ones are attributed to the negative class. However, if the binary classification involves, e.g., two breeds or sexes, sensitivity and specificity are only conventional names, the first of which indicates individuals of one breed, and the second refers to those from another class [136].

Sensitivity (true positive rate, *TPR*) is given by the following formula [97,138]:

(18) $$TPR=\frac{TP}{TP+FN}.$$

This is the ratio of the number of positive class individuals correctly classified by the model to the number of all individuals actually belonging to the positive class. The higher the sensitivity, the better the model’s ability to identify individuals (elements) belonging to the positive class (e.g., mastitic cows) [136,139].

Specificity (true negative rate, *TNR*) can be expressed as [8,138]

(19) $$TNR=\frac{TN}{TN+FP}.$$

This is the ratio of the number of negative class individuals correctly classified by the model to the number of all individuals actually belonging to the negative class. The higher the specificity, the greater the ability of the model to indicate individuals (elements) belonging to the negative class (e.g., healthy cows) [136,139].

Accuracy (*Acc*) is the percentage of correctly classified cases from both [positive (*TP*) and negative (*TN*)] classes [97,137,138]:

(20) $$Acc=\frac{TP+TN}{TP+TN+FP+FN}.$$

In the case of a larger number of classes (more than two), performance indicators are calculated separately for each class, treating all other classes as a so-called meta-class. Indicators calculated in this way can be averaged for all classes using micro- or macro-averaging [140,141].

Below is an example of a confusion matrix for three classes and a method for calculating sensitivity and specificity (Table 2).

Sensitivity for Class A:

(21) $${TPR}_{A}=\frac{{TP}_{A}}{{TP}_{A}+{FN}_{A/B}+{FN}_{A/C}}.$$

Sensitivity for Class B:

(22) $${TPR}_{B}=\frac{{TP}_{B}}{{TP}_{B}+{FN}_{B/A}+{FN}_{B/C}}.$$

Sensitivity for Class C:

(23) $${TPR}_{C}=\frac{{TP}_{C}}{{TP}_{C}+{FN}_{C/A}+{FN}_{C/B}}.$$

Specificity for Class A:

(24) $${TNR}_{A}=\frac{{TN}_{A}}{{TN}_{A}+{FP}_{A}},$$

where: *TN_A_* = *TP_B_* + *TP_C_*, *FP_A_* = *FN_B/A_* + *FN_C/A_*.

Specificity for Class B:

(25) $${TNR}_{B}=\frac{{TN}_{B}}{{TN}_{B}+{FP}_{B}},$$

where *TN_B_* = *TP_A_* + *TP_C_*, *FP_B_* = *FN_A/B_* + *FN_C/B_*.

Specificity for Class C:

(26) $${TNR}_{C}=\frac{{TN}_{C}}{{TN}_{C}+{FP}_{C}},$$

where *TN_C_* = *TP_A_* + *TP_B_*, *FP_C_* = *FN_C/A_* + *FN_B/C_*.

Other predictive performance measures that are not so frequently used in model description include positive and negative predictive values and false positive and false negative rates.

Positive predictive value (*PPV*) is the ratio of all positive classifications made by the model (correctly assigned to the positive class) to all individuals or elements (correctly or incorrectly) assigned by the model to the positive class [137,138]:

(27) $$PPV=\frac{TP}{TP+FP}.$$

Negative predictive value (*NPV*) is the ratio of all negative classifications made by the model (correctly assigned to the negative class) to all individuals or elements assigned by the model to the negative class [137,138]:

(28) $$NPV=\frac{TN}{TN+FN}.$$

False negative rate (*FNR*) or miss rate indicates the ratio of false negative cases (misclassified by the model into the negative class but actually belonging to the positive class) to all cases in the positive class [139]:

(29) $$FNR=\frac{FN}{TP+FN}=1-TPR.$$

False positive rate (*FPR*) or fall-out shows the ratio of false positive cases (misclassified by the model into the negative class) to all cases in the negative class (the so-called false alarm) [138,142]:

(30) $$FPR=\frac{FP}{TN+FP}=1-TNR.$$

Sensitivity and specificity are in principle independent of each other; however, an increase in sensitivity often results in a decrease in model specificity. On the other hand, *PPV* and *NPV* depend on each other. Increasing *TP* decreases *FN* and vice versa, which affects the values of *PPV* and *NPV*. A summary of these performance indicators for a multi-class classification case is presented in [143].

Sometimes, the so-called *F*_1_ ratio is used to describe model quality [138,142]:

(31) $$F_{1}=\frac{2\cdot TP}{2\cdot TP+FP+FN}.$$

*F*_1_ indicates the accuracy with which the model captures relevant processes and accounts for class imbalance, focusing on the proportion of positive predictions and actual positive cases. This indicator tries to balance sensitivity and precision to find the best compromise between them. The more unbalanced the data set, the lower the *F*_1_ score, even with the same overall accuracy [144,145].

The measure that maximizes sensitivity, specificity, and accuracy at the same time is the Matthews correlation coefficient (*MCC*) [8,146,147]:

(32) $$MCC=\frac{TP\cdot TN-FP\cdot FN}{\sqrt{\left(TP+FP)\cdot (TP+FN)\cdot (TN+FP)\cdot (TN+FN\right)}}.$$

*MCC* ranges from −1 to +1, where +1 corresponds to perfect classification, −1 indicates completely wrong predictions, and values around 0 show random classification [145].

Cohen’s kappa coefficient (*κ*) can also be used in some situations to describe model performance [146]:

(33) $$\kappa =\frac{2(TP\cdot TN-FN\cdot FP)}{\left(TP+FP\right)\left(FP+TN\right)+\left(TP+FN\right)(FN+TN)}.$$

It ranges from +1, which indicates perfect agreement between evaluators, to −1, which means that evaluators select different labels for each case. A value of 0 shows that the agreement is merely due to chance. An example of the use of such a coefficient in dairy cattle research can be found in [148], where within-cow changes in visual rumen fill scores were evaluated to estimate dry matter and feed intake, or in [149], where Cohen’s kappa coefficient was applied to assess inter-rater score agreement on teat swab images, and the intraclass correlation coefficient was utilized to evaluate both intra-rater score agreement and machine reliability.

A measure that assesses the similarity between two categories is the Dice–Sorensen coefficient (*DSC*) [8,150]:

(34) $$DSC=\frac{2\left|A\cap B\right|}{\left|A\right|+\left|B\right|},$$

where *A* is the number of category *A* elements and *B* is the number of category *B* elements.

It is useful for imbalanced data sets and is commonly applied in image segmentation tasks, natural language processing, and other fields that require the measurement of similarity between two sets [151]. *DSC*, ranging between 0 and 1, can be represented using a classification matrix [152,153]:

(35) $$DSC=\frac{2TP}{2TP+FP+FN},$$

similar to the *Jaccard index*, which also measures the similarity between two sets [8,154]:

(36) $$Jaccard\;index=\frac{TP}{TP+FP+FN}.$$

An example of the use of this index, among others, can be found in [155], in which the bacterial profiles of milk samples taken from healthy cows and those with clinical and subclinical mastitis were analyzed.

### 2.14. Receiver Operating Characteristic (ROC) Curve and Area Under the Curve (AUC)

**Figure 7:**
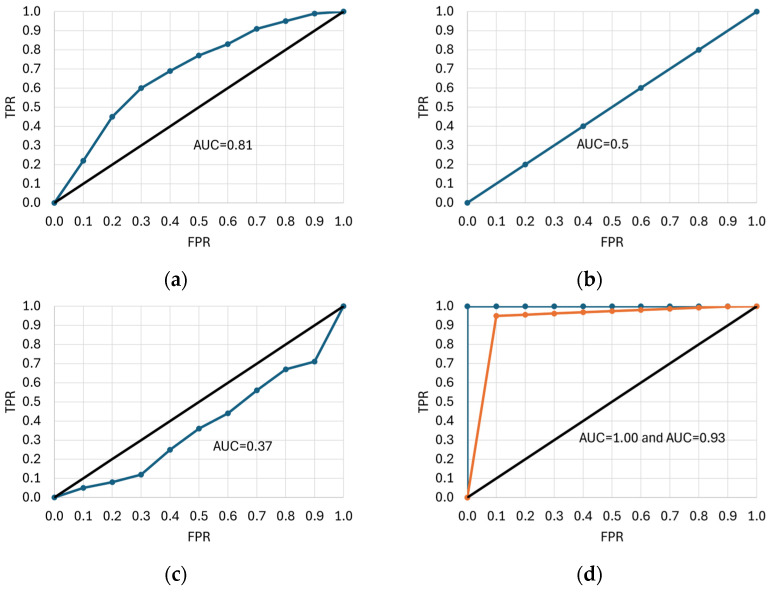

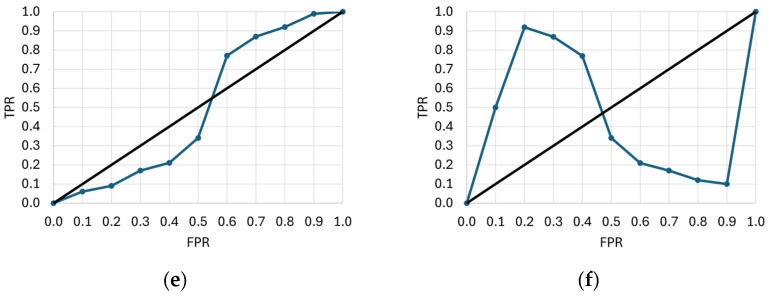
Examples of receiver operating characteristic (ROC) curves. *TPR*—true positive rate, *FPR*—false positive rate, AUC—area under the curve. Different ROC curve shapes (**a**–**f**) are explained in the text.

The ROC curve is a two-dimensional graph with *FPR* on the *x*-axis and sensitivity (*TPR*) on the *y*-axis. It is created by calculating the *TPR* and *FPR* for each decision threshold of the classification model and plotting the points (*FPR* and *TPR*) on a graph [74,156,157]. This curve makes it easier to quantify the extent of class separability and the data quality required to accurately distinguish between predicted objects. The area under the ROC curve, or AUC-ROC, is often used as a performance measure for a classification model [8]. Figure 7 shows six examples of ROC curves. The ROC curve lying above and to the left of the *y* = *x* diagonal indicates a more deterministic model, i.e., *TPR* approaches unity, and AUC increases (Figure 7a, AUC = 0.81). On the other hand, the curve coinciding with the *y* = *x* diagonal corresponds to *TPR* = 0.5 and a random model (Figure 7b, AUC = 0.5), whereas the ROC curve below the diagonal (Figure 7c, AUC = 0.37) denotes a less deterministic model (but may also result from improper class encoding) [158,159,160]. The AUC values close to unity indicate a very good classifier (Figure 7d, AUC = 1 and AUC = 0.93), but such a situation occurs quite rarely in practice, and the shape of the curve is usually less optimal. In some cases, the ROC curves shown in Figure 7e,f can be obtained if the distinguished category (e.g., ketosis in cattle) corresponds to both high and low values of the predictor variable (or vice versa; e.g., when deviations from the norm are detected). The AUC does not differentiate between the significance of errors in the different parts of the ROC curve, which can lead to situations where the classifier, despite its high AUC, is not optimal for applications that require fast and accurate detection of outliers. Additionally, the costs associated with false positives and false negatives can significantly affect user preferences. For example, if a classifier incorrectly assigns a negative case to a positive class, a healthy cow may be misclassified as an ill one. This results in unnecessary treatment, medication use, taking the animal out of service, and veterinary costs. Conversely, when an ill cow is incorrectly classified as healthy, complications, longer treatment duration, reduced productivity, economic losses, or even the death of the animal can occur.

Sometimes, it is necessary to decide whether *FP* or *FN* is more important. In the case of *FP*, a model that does not incorrectly assign an object from a negative class to a positive one is better, even at the expense of assigning too many cases from a positive class to a negative one. If *FN* is more important, a model with a lower probability of assigning an object from a positive class to a negative class is preferred. The shape of the ROC curve can be helpful in making the right decision [156,161].

AUC-ROC has become a commonly used metric for evaluating binary classification tasks in most scientific fields. It ranges from 0 (the worst performance) to 1 (excellent performance) [147]. However, this metric is based on predictions that may have insufficient sensitivity and specificity, and says nothing about the *PPV* (also known as precision) or *NPV* achieved by the classifier, potentially generating overly optimistic results [137]. Since the AUC-ROC is often considered alone (without precision or *NPV*), it may indicate that the classifier is successful, which may not be true. A given point in the ROC space does not identify a single confusion matrix or a group of matrices with the same *MCC* value. Sensitivity and specificity can cover a wide range of *MCC*, undermining the reliability of the AUC-ROC as a measure of model performance [147,157]. In contrast, *MCC* assumes high values in the [−1; +1] range only if the classifier achieves good results for all four metrics in the confusion matrix: sensitivity, specificity, precision, and *NPV*. High *MCC* always corresponds to high AUC-ROC, but not vice versa, which is why Chicco et al. [137] postulate that *MCC* should be used instead of AUC-ROC. Other authors have proposed some improvements in the application of the ROC curve and ROC-AUC by highlighting important threshold points on the curve, interpreting the shape of the curve, defining lower and upper limits for ROC-AUC, or mapping occurrence density in each interval of the ROC curve [162].

### 2.15. Model Development

The use of ML in precision livestock farming requires the collection of a large amount of data via sensors and other technical devices [2]. The data collected in this way can be inaccurate, may not reflect the actual state of both the phenomenon under study on a given farm, or may represent only an incidental fragment recorded at a specific time [163]. This leads to errors, i.e., the results obtained from the calculations may be incorrect. Consequently, poor decisions are made to the detriment of the animals and the farmer. It is certainly a challenge for the breeder or the person in charge of the calculations to have some sort of contingency plan in case various anomalies occur [164].

The development of different ML models requires adequate training data. If they are not representative of the problem being solved, the results generated by the model become inaccurate [165]. The data used in dairy farming can be acquired from, e.g., system records, current measurements, different sensors, etc. Such a huge collection of data is not immediately applicable, and pre-processing becomes necessary. Therefore, a process of data analysis involves three main steps: data pre-processing, actual data exploration, and so-called post-processing [166]. At the pre-processing stage, a dataset is prepared for analysis so that the data categories appropriate for problem solving are identified and selected. Pre-processing involves data filtering, selection of data types and variables, data cleaning and transformation, and final data compilation in a format appropriate for a specific ML algorithm [70].

Many of the datasets received for analysis at the initial stage of the data exploration process exist in raw, unprocessed, and insufficiently described form. Therefore, it is vital to identify data categories and relationships between them and to select variables describing objects and determine associations among them [167]. The most common types of variables are numeric and nominal. Sometimes, character strings are converted into numeric form. Data cleaning, on the other hand, involves removing missing, erroneous and inconsistent values or outliers [168]. Missing data can be replaced with new values, noisy data may be de-noised, and outliers must be identified and removed.

Data transformation includes smoothing, variable generation, aggregation, normalization, discretization, and concept hierarchy creation for nominal variables. It can significantly increase model accuracy and make the data more suitable for ML methods (e.g., by projecting and transforming variables with multiple categories into binary form) [167]. The selection and development of an appropriate model must be oriented toward the specific research problem. In the case of quantitative variables (e.g., milk yield, body weight, fat content, etc.), a model most suitable for regression problems is probably required [169]. In the case of nominal variables with a limited number of potential values (the so-called categories or classes, e.g., disease occurrence, successful conception, etc.), a model best suited for classification problems is preferred. It is equally important to optimize the model’s hyperparameters (hyperparameter tuning) and implement them in practice (model deployment). A comprehensive review of this issue is provided in [142].

### 2.16. Dimensionality Reduction

If the analyzed data set contains conditional variables that do not contribute much to solving the problem, redundant variables, or those characterized by collinearity (or another form of dependence), their reduction can limit the set of variables to those that best explain the phenomenon under study (the decision variable). This, in turn, may improve predictions by removing irrelevant variables, simplifying the model, increasing its performance, and facilitating the visualization of the data structure [5].

Reducing the number of variables in a data set, while it may degrade accuracy, makes exploration, visualization, and the entire data analysis process easier and faster for ML algorithms [170]. A commonly used method is principal component analysis (PCA), which reduces dimensionality by transforming a large set of variables into a smaller one that still contains most of the information from the original set [167]. PCA primarily involves standardizing a range of continuous initial variables so that each of them contributes equally to the analysis, calculating covariance matrices to identify correlations between variables, determining eigenvectors and eigenvalues of covariance matrices to identify principal components, creating feature vectors to select principal components, and transforming the data along the principal component axes [171,172]. The new variables (principal components) are the uncorrelated linear combinations or mixtures of the initial variables. PCA attempts to contain the maximum possible variance in the first component, followed by the maximum remaining variance in the second component, and so on [173]. Unfortunately, the principal components formed in PCA are more difficult to interpret, since they are the linear combinations of the initial variables [174].

An example of the use of PCA in dairy cattle farming can be found in [175], where this method was applied to identify a more suitable and accurate set of features to predict the total milk yield in cows and obtain increased economic return without the problem of collinearity. On the other hand, Wang et al. [98] used PCA to obtain estrus indicators in dairy cows based on behavioral indices generated from collected data (standing, lying, walking, feeding, and drinking time; switching time between activity and lying; steps; displacement; and average velocity). Estrus detection was performed using k-NN, ANN, linear discriminant analysis, and CART. PCA was also applied for the early detection of mastitis and lameness. It extracted uncorrelated principal components by linearly transforming the raw data so that the first few PC contained most of the variation in the original dataset. Milk yield and conductivity and feeding events (feed consumption, number of feeding visits, and time at the trough) were used to identify mastitis, whereas pedometer activity and feeding patterns were utilized to detect lameness [176].

### 2.17. Multimodal Learning and Data Fusion

Multimodal learning is an example of an ML application using different types of data (modalities) simultaneously. In this case, the AI system learns from multiple sources of information (text, audio, video, motion sensors, etc.) at the same time. In reality, information rarely comes in one form. When observing a cow, one also hears the sounds it makes, sees the way it moves, or has access to its health and performance records.

**Figure 8:**
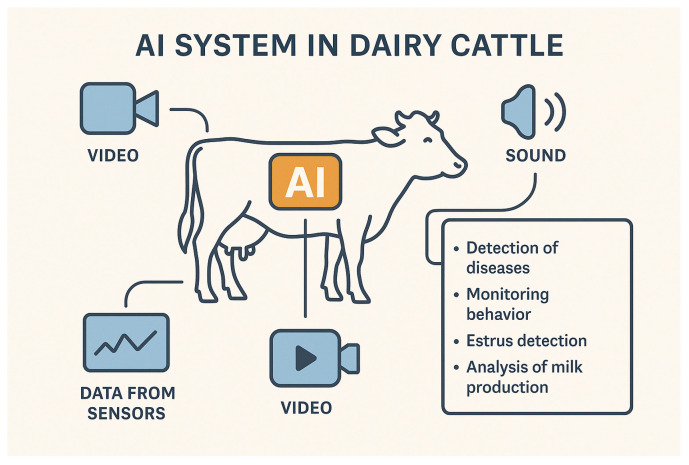
Diagram of the use of different information types for a comprehensive analysis of the cow.

Unlike traditional AI models, which are typically designed to handle only one type of data, multimodal AI combines and analyses their different forms to achieve a more comprehensive understanding of the problem at hand and generate more reliable results. For example, when analyzing a specific cow, multimodal systems attempt to mimic the human ability to combine information from multiple sources in order to better understand the context, become more precise, and resemble the human approach to the same task (Figure 8).

In dairy farming, such applications may relate to early disease detection [AI simultaneously analyses cow’s images, movements, sounds (e.g., coughing), and sensor data (e.g., temperature, steps, and heart rate), thus detecting early signs of the diseases such as mastitis, respiratory infections, or lameness]. Camera images, data from the GPS collars, and stall sensors provide additional information on the amount of time the cow spends lying down, walking, feeding, etc., making it possible to quickly detect stress, pain, or illness if the habits suddenly change. The recording of the number of steps, images of cow movement, body temperature changes, or the analysis of body secretions may simultaneously help to optimize the timing of insemination (based on oestrus detection). In milk production systems, AI determines the effect of different factors (e.g., nutrition, season, and health status) on milk yield, which improves diet formulation and rearing conditions for each animal (i.e., precision breeding). AI can also learn to “read” a cow’s body language in order to recognize pain or stress, which are often difficult for humans to perceive. By combining genetic and health data, behavioral and production traits, AI may help select the best animals for further breeding.

A specific example of multimodal AI application is veterinary diagnostics, where instant access to vast amounts of information supports veterinarians in making evidence-based decisions. The ability of AI systems to integrate different types of data, including medical imaging (e.g., X-ray and computed tomography scans), textual data (e.g., clinical notes and laboratory reports), behavioral videos (e.g., gait analysis), and audio recordings (e.g., breathing sounds) provides powerful diagnostic capabilities and assists veterinarians in their daily work. By correlating inputs, multimodal AI facilitates a more holistic understanding of the animal’s condition, increases diagnostic accuracy, and improves decision-making efficiency [177].

Vu at al. [178] presented a multimodal dataset for dairy cattle monitoring, which contains a large amount of synchronized, high-quality measurements related to behavioral, physiological, and environmental factors. Biweekly data collected using wearable and implantable sensors deployed on dairy cows included 4.8 million frames of high-resolution image sequences from four isometric cameras, temperature and humidity records from environmental sensors, as well as milk yields and outdoor weather conditions. The availability of such a collection and its comparative tests could facilitate research into multimodal cow monitoring.

Russelo et al. [179] developed a multimodal model based on video sequences and temporal information for more stable tracking of cow postures (especially important in lameness detection), while Afridi et al. [180] investigated the effect of different data types (RGB color, depth, and segmentation) on the accuracy of cow weight estimation, thus presenting the application of multiple imaging modalities in practice. Finally, Themistokleous et al. [181] demonstrated a multi-modal approach (image-processing DNN) for combining ultrasound data with udder condition observations to classify the level of milk production.

An example of combining information from multiple sources is shown in Table 3.

Linked to the concept of multimodal learning is a fusion dataset, which contains information from multiple sources (modalities) that can be integrated for a more accurate analysis by AI models. ‘Fusion’ in this case means combining information from different sources into a coherent whole. In the context of AI and ML (especially multimodal learning), this could mean, e.g., combining camera images with microphone sounds for their joint analysis. Another example is integrating text and sensor data to better understand the context or using GPS coordinates and accelerometer readings to analyze movement and location together. Fusion data may include video footage of the cow, the sounds it makes, features from the sensor collar, or production records (milk yield, lactation, and temperature). All this information is collected simultaneously and synchronized to train AI models that are able to predict disease occurrence, detect heat, or assess welfare.

In a nutshell, multimodal learning is a technique that uses multiple models to improve the final results, and dataset fusion is the process of combining different data types into a single dataset for model training or information processing. Table 4 shows an example of the dataset records for heat detection in dairy cows.

### 2.18. Trends of ML Use in Dairy Cattle Farming

Finally, Table 5 summarizes the ML models most frequently used in dairy cattle farming for the years 2020–2024. The search query included two terms (“full model name” and “dairy cattle”) and was submitted to the Google Scholar, Web of Science, and Scopus databases in February 2025. The first of the databases made it possible to search article full texts, whereas the two remaining ones limited the results to the title, abstract, and keywords. It should be noted that Web of Science and Scopus rank publications according to a set of criteria, and the number of articles in these databases is significantly lower compared with Google Scholar, which also searches for books, conference proceedings, and dissertations. The numbers presented in Table 5 are only a reference point for readers (application popularity ranking), since they are excessively high for some models due to the application of several methods in the same study.

Of the models presented, LR has been used most frequently in different studies involving dairy cattle. LogR was also very popular, which is especially evident for Google Scholar. The third position in the ranking was occupied by CA according to Google Scholar and RF according to Web of Science and Scopus. Relatively low interest has been exhibited in GMM (Google Scholar), SVM and NBC (Web of Science), as well as NBC and CHAID (Scopus).

Table 6 shows the 27 most cited articles that used ML methods in dairy cattle farming (according to the Web of Science Core Collection and other Web of Science databases). The search query [ALL = (“machine learning”) AND ALL = (“dairy cattle”)] was limited to the period 2020–2024. Out of 30 initially found papers, three articles were excluded from this collection since they were unrelated to the subject matter (studies on plants, pigs, or beef cattle).

The most cited article had 80 citations in the Web of Science Core Collection database and 91 in the other Web of Science databases, while the least popular article in the list had 18 citations in each of the sources.

## 3. Conclusions

The development of artificial intelligence is providing researchers and breeders with a variety of tools for the studies associated with dairy cattle farming, including genetic, environmental, and behavioral factors. In the analysis of large datasets, the use of some ML methods is essential. In this review, we presented various examples of ML applications in dairy cattle breeding and husbandry, mainly in classification and regression, and the methods for their evaluation. Many researchers have applied ML models, which perform better or worse in different tasks. These models show great potential for pattern recognition from large and noisy datasets. However, it is difficult to identify the best criterion for selecting the right model for a problem to be solved and to interpret the results obtained. The popularity of various ML models in dairy cattle farming has remained somewhat constant for most of them in recent years, and it is currently hard to predict in which direction the field will evolve in the future. Will the interest in ML begin to flag (as in the case of neural networks in the past), only to grow again later? However, when developing models that would predict the future perfectly, we should not forget that this can transform into a chase for Laplace’s demon.

## Figures and Tables

**Table 1:** Confusion matrix.

Predicted Values	Real Values	
	Positive Class	Negative Class
Positive class	True Positive (*TP*)	False Positive (*FP*)
Negative class	False Negative (*FN*)	True Negative (*TN*)

**Table 2:** Confusion matrix for three classes.

Predicted Values	Real Values		
	A	B	C
A	*TP_A_*	*FN_B/A_*	*FN_C/A_*
B	*FN_A/B_*	*TP_B_*	*FN_C/B_*
C	*FN_A/C_*	*FN_B/C_*	*TP_C_*

**Table 3:** Examples of data fusion applications in dairy cow monitoring and management.

Area of Application	Modalities	Example
Sound analysis	Acoustics + natural language processing	Kate and Neethirajan [182]
Physiological-behavioral monitoring	Video + ultrawideband + sensors	Vu et al. [178]
Lameness/position assessment	Video + temporal data	Rusello et al. [179]
Body weight estimation	RGB + depth + segmentation	Afridi et al. [180]
Milk production	Ultrasound imaging + deep learning	Themistokleous et al. [181]
Influence of genes on milk production and cow health	Genetic and phenotypic data	Gutiérrez-Reinoso et al. [183]
Using sensors to track activity and temperature, combined with production data	Data from health and production monitoring systems	Ferreira et al. [184]
Impact of weather conditions on milk yield	Environmental and production data	Li et al. [185]
Using laboratory test results with data on supplementation and rearing	Laboratory and management data	Mota et al. [186]

**Table 4:** Data fusion—example records for one cow (one day or one observation).

Cow_ID	Timestamp	Temp (°C)	Steps No.	Rumination Time (min)	Moo Frequency (Hz)	Moo Type	Video Posture	Milk Yield (l)	Heat Indicator
235	22 May 2024 08:00	38.4	472	510	350	“distress”	“lying”	29.3	0.81
235	22 May 2024 20:00	38.9	215	430	410	“normal”	“standing”	28.5	0.97

Cow_ID: the unique identifier for each cow; Timestamp: the time when the measurement was taken; Temp (°C): cow’s body temperature measured with a sensor placed on her neck or vulva; Steps No.: the number of cow’s steps recorded by a collar or leg accelerometer; Rumination Time: the duration of chewing activity, tracked by a rumination sensor like a bolus or collar; Moo_Frequency: the average sound frequency (Hz) of the cow’s vocalizations, captured by a microphone; Moo_Type: the classification of the cow’s vocalization type, analyzed through natural language processing or acoustic analysis; Video_Posture: the cow’s posture (e.g., lying down or standing), determined by a camera with computer vision; Milk_Yield: the amount of milk produced, measured by an automatic milking system (liters); Heat_Indicator: the probability of the cow being in heat (ranging from 0 to 1), calculated by a multimodal model.

**Table 5:** ML models most commonly used in dairy farming between 2020 and 2024.

Model	Google						Web of Science						Scopus					
	2020	2021	2022	2023	2024	Total	2020	2021	2022	2023	2024	Total	2020	2021	2022	2023	2024	Total
LR	1320	1580	1630	1550	1570	7650	22	29	21	22	26	120	27	40	23	29	30	149
LogR	909	1070	1150	1100	1110	5339	39	58	38	43	48	226	45	72	68	60	74	319
ANN	358	513	689	764	823	3147	1	2	4	2	2	11	8	10	11	14	12	55
CA	385	496	458	470	482	2291	6	6	6	4	9	31	14	17	8	11	17	67
RF	223	309	407	468	550	1957	8	8	14	12	11	53	9	14	16	27	22	88
SVM	158	194	290	274	301	1217	0	0	0	0	0	0	6	9	3	15	9	42
Adaboost	27	36	58	63	55	239	1	0	1	1	1	4	1	0	0	1	1	3
k-NN	34	40	74	66	113	327	0	0	2	2	0	4	2	4	4	5	4	19
NBC	27	33	51	48	51	210	0	0	0	0	0	0	0	1	0	0	0	1
CART	24	34	44	36	39	177	0	1	0	0	0	1	1	2	2	0	0	5
CHAID	10	7	26	17	21	81	0	0	1	1	0	2	0	0	0	1	1	2
GMM	1	9	2	3	3	18	1	1	2	0	0	4	1	0	3	0	0	4
MARS	1	1	3	3	6	14	1	0	2	1	1	5	0	0	2	0	2	4

LR—linear regression, LogR—logistic regression, CA—cluster analysis, RF—random forest, ANN—artificial neural network, SVM—support vector machine, k-NN—k-nearest neighbor, NBC—naive Bayes classifier, CART—classification and regression tree, MARS—multivariate adaptive regression spline, CHAID—chi-squared automatic interaction detection, GMM—Gaussian mixture model.

**Table 6:** Summary of the most cited articles on the use of machine learning in dairy cattle farming.

Rank	Ref.	Method	Application Field	Number of Citations	
				Web of Science Core Collection	Remaining Web of Science Databases
1	[187]	RF	Classification of the microbiome on the basis of rumen metabolites	80	91
2	[188]	Hybrid clustering and classification model (RF, k-NN)	Lameness detection	72	75
3	[140] *	Deep learning (Mask R-CNN, Faster R-CNN, YOLO v3 and v4, DeepLab v3, U-Net, ResNet, Inception, Xception, and VGG16)	Computer vision for animal identification and behavior, feed intake, animal body weight, and others	71	80
4	[189]	Stacking ensemble learning framework (SELF), support vector regression (SVR), kernel ridge regression (KRR) and elastic net (ENET), genomic best linear unbiased prediction (GBLUP), BayesB	Predicting genomic estimated breeding values	42	48
5	[190]	RF	Mastitis detection	39	41
6	[191]	LogR, Gaussian naïve Bayes, RF	Heat stress prediction	36	42
7	[192] *	Partial least squares, ANN, SVM, Bayes B, LR, principal components regression	Prediction of milk composition, feed efficiency, methane emission, fertility, energy balance, health status and meat quality traits from infrared spectrometric data	35	39
8	[193]	Long-term recurrent convolutional networks	Monitoring the activity and social behavior in cows	34	35
9	[194]	Long short-term memory (LSTM) network, gated recurrent unit (GRU), bidirectional LSTM (BLSTM), and stacked LSTM	Monitoring of cow body temperature	34	42
10	[195]	RF, k-medoids algorithm	Analysis of cattle behavior (grazing, ruminating, laying and steady standing)	30	32
11	[196]	BLSTM, LSTM, RUSBoosted tree	Predicting calving date and the eight-hour period before calving	29	33
12	[197]	RF	Modelling the milk yield of cows under heat stress conditions	28	30
13	[198] *	ML in general	Targeted reproductive management based on genomic predictions; analysis of behavioral, physiological, and performance parameters, based on individual cow and herd performance records	27	31
14	[199] *	ML in general	The future of phenomics in the rearing and breeding of cattle	27	31
15	[200]	LR, partial least squares regression, ANN, and stacked ensembles	Predicting feed intake and residual feed intake based on behavioral and metabolic data in addition to classical performance variables	25	29
16	[201]	Decision trees, SVM, PCA	Identification of candidate genes and functional modules associated with mastitis	24	24
17	[202]	RF, gradient boosting machine (GBM), penalized regression, partial least squares (PLS) regression	Prediction of difficult-to-measure traits in Holstein cattle based on milk infrared spectral data	24	26
18	[203] *	Linear regression, LogR, SVM, Fuzzy C-mean (FCM), ANN, CART, CNN, RF, threshold discrimination, YOLO, histogram oriented gradient (HOG), fuzzy logic	Use of infrared thermography to assess the health of cows (mastitis, lameness, respiratory diseases, physiological characteristics, stress, temperament, oestrus)	23	23
19	[204] *	JRip, J48, RF, ANN, penalized linear regression, gradient boosted machines, Mask R-CNN, generalized additive model	Analysis of heat stress in cows	20	20
20	[205] *	ML in general	Analysis of the heat stress response in cattle	19	19
21	[206]	Stacking ensemble learning including elastic net (EN), gradient boosting machine (GBM), extreme gradient boosting (XGBoost), and ANN	Predicting cheese quality related traits in dairy cows	19	22
22	[207]	YOLOv2 COMV	Digital dermatitis detection based on camera images	19	20
23	[208]	Mask R-CNN	Determination of pixel-level segmentation masks for the cows in the video material	19	19
24	[209]	XGBoost	Predicting lameness in cattle	18	20
25	[21]	Generalized Linear Models (GLM), ANN, RF	Predicting oestrus in heifers based on feeding behavior	18	20
26	[210]	Catboost, AdaBoost, RF, linear regression, decision trees, adaptive boosting, SVM	Predicting body weight of dairy cattle from 3D images	18	18
27	[122]	YOLO, support vector regression (SVR), k-NN, RF, linear regression, polynomial regression	Monitoring and predicting the body temperature of cattle	18	18

* Review, LR—linear regression, LogR—logistic regression, RF—random forest, ANN—artificial neural network, SVM—support vector machine, k-NN—k-nearest neighbor, CART—classification and regression tree, CNN—convolutional neural network.

## Data Availability

Not applicable.

## Abbreviations

The following abbreviations are used in this manuscript:

LR	Linear regression
LogR	Logistic regression
CA	Cluster analysis
RF	Random forest
ANN	Artificial neural network
SVM	Support vector machine
k-NN	K-nearest neighbor
NBC	Naive bayes classifier
CART	Classification and regression tree
MARS	Multivariate adaptive regression spline
CHAID	Chi-squared automatic interaction detection
DNN	Deep neural network
CNN	Convolutional neural network
GMM	Gaussian mixture model
